# *Artyfechinostomum sufrartyfex* Trematode Infections in Children, Bihar, India

**DOI:** 10.3201/eid2508.181427

**Published:** 2019-08

**Authors:** Yugal K. Prasad, Suman Dahal, Barsha Saikia, Bobita Bordoloi, Veena Tandon, Sudeep Ghatani

**Affiliations:** Shri Shubh Lal Hospital and Research Centre, Sitamarhi, India (Y.K. Prasad);; Sikkim University, Gangtok, India (S. Dahal, B. Saikia, B. Bordoloi, S. Ghatani);; Biotech Park, Lucknow, India (V. Tandon)

**Keywords:** echinostomiasis, trematodiasis, metacercaria, Artyfechinostomum sufrartyfex, trematodes, infections, Pila globosa, snails, molecular markers, DNA barcoding, food safety, parasites, vector-borne infections, children, Bihar, India

## Abstract

Eating raw or insufficiently cooked mollusks is a known risk factor for human echinostomiasis. We confirmed identification of *Artyfechinostomum sufrartyfex* trematodes as the causative agent of disease among 170 children in northern Bihar, India. We also identified the snail *Pila globosa* as a potential source of infections in the study area.

Foodborne intestinal trematodiasis, especially that caused by members of the family *Echinostomatidae*, is an emerging yet neglected public health disease. Approximately 24 echinostome species cause human echinostomiasis and are highly endemic to Southeast Asia and the Far East; major foci are located in China, India, Indonesia, South Korea, Malaysia, the Philippines, and Thailand (*1*).

Previously, only 2 deaths attributed to the echinostomid fluke *Artyfechinostomum sufrartyfex* were reported from the states of Assam and Tamil Nadu in India (*2*,*3*). During 2004–2017, several cases of echinostome infection were reported in children at Shri Shubh Lal Hospital and Research Centre in Bihar, India. 

## The Study

This study was approved by the Institutional Ethics Committee of Sikkim University (SU/IEC/2017/04), Gangtok, India. A total of 170 cases of *A. sufrartyfex* trematode infection occurred in northern Bihar, India, mostly in children <12 years of age. The children lived in the districts of Sitamarhi and Sheohar in the state of Bihar. Signs and symptoms were diarrhea (persistent/chronic and acute) with watery or mucus-bound stool, vomiting, loss of appetite, weakness, passage of red worms in stool or vomit, swelling of the feet and the entire body, fever, cough, breathlessness, night blindness, and urticarial rashes (Table 1).

**Table 1:** Clinical signs and symptoms for 170 patients with *Artyfechinostomum sufrartyfex* trematode infections, Bihar, India

Sign or symptom	No. positive
Persistent/chronic diarrhea	150
Acute diarrhea	15
Vomiting	153
Passage of red worms in stool/vomit	49
Fever	67
Loss of appetite	169
Weakness	169
Cough	35
Breathlessness	15
Swelling of feet and entire body	78
Night blindness	7
Rashes	2

Physical examination showed that most patients were anemic. Clinical laboratory investigations showed leukocytosis and eosinophilia. However, systemic examination showed no adverse effects of the cardiovascular, abdominal, and central nervous systems (Table 2). Levels of serum alanine aminotransferase, bilirubin, blood urea, creatinine, electrolytes, sodium, potassium, and chloride were within reference limits.

**Table 2:** General and systemic examination results for 170 patients with *Artyfechinostomum sufrartyfex* trematode infections, Bihar, India

Characteristic	No. positive
Anemia	170
Fever	8
Some dehydration	129
Severe dehydration	41
Malnutrition grading*	
I	11
I (K)	10
II	38
II (K)	20
III	31
III (K)	28
IV	15
IV (K)	22
Not grouped	1
No malnutrition	6
Pedal and generalized edema	82
Glossitis	6
Angular stomatitis	4
Xerosis conjunctiva	8
Urticaria (rashes)	2

*Grading system of the Indian Academy of Pediatrics (Mumbai, India) for protein energy malnutrition based on a weight for age standard. K, Kwashiokor (patient has edema); I, mild; II, moderate; III, severe; IV, very severe.

These children were immediately hospitalized and kept under careful observation with routine monitoring of stool and vomit for worms. Once worms were observed in samples, the patients were given praziquantel (75 mg/kg in 3 divided doses orally for 2 days) and monitored. At administration of the drug, patients started passing more worms in stool. We recovered >50 worms but <300 worms from each child patient. The infection subsided after the standard dose of praziquantel, and most patients recovered from the infection.

However, we observed 11 deaths: 2 patients each during 2004, 2007, 2008, 2012, and 2013 and 1 patient during 2009. Severe acute malnutrition with or without edema and large numbers of worms were major clinical conditions observed for these deaths. Nine children had persistent diarrhea with severe dehydration and shock, and 2 of them had acute diarrhea, severe dehydration, and shock.

**Figure 1:**
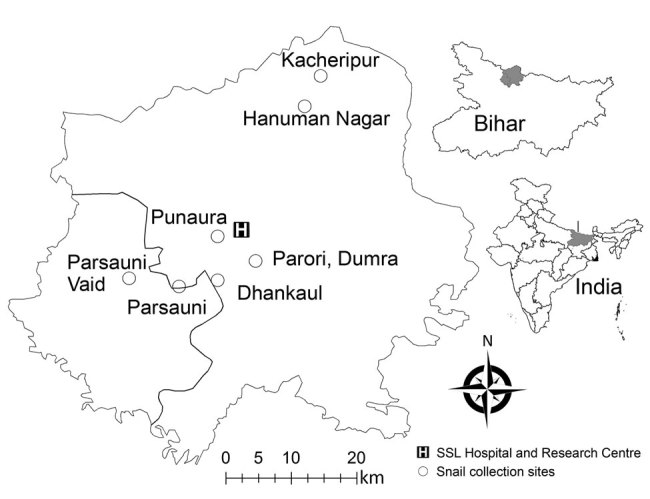
Collection sites of clinical samples from patients infected with *Artyfechinostomum sufrartyfex* trematodes at SSL Hospital and Research Center, Sitamarhi, Bihar, India. Black border indicates district boundary. Insets show location of Sitamarhi in Bihar and location of Bihar in India. SSL, Shri Shubh Lal.

The infected patients frequently consumed raw snails. The most prevalent snail species in the study areas was *Pila globosa*, which the children collected from the banks of ponds/ditches and waterlogged paddy fields grossly contaminated with human and animal excreta (168/170 cases, 99%). Therefore, we surveyed as many as 8 sites in 2 districts (Sitamarhi and Sheohar) for snail samples from their natural habitats (Figure 1).

We screened the snails by using a digestion technique with a 0.5% pepsin/0.1% HCl solution and found that the snails were heavily infected with metacercariae, which are the encysted infective stage of the trematode. The prevalence of metacercariae in the snails ranged from 16.12% in Hanumannagar to <48.19% in Punaura (Appendix).

**Figure 2:**
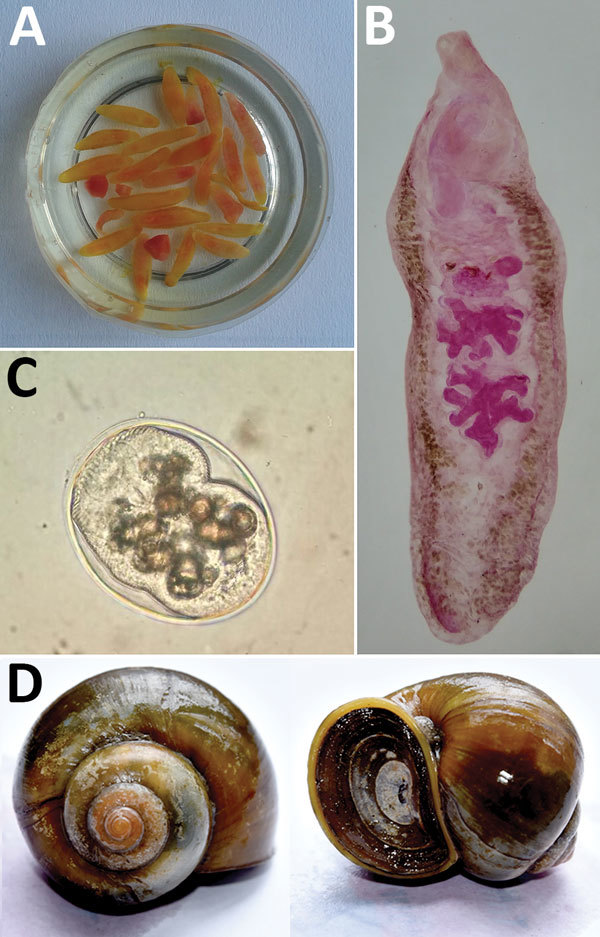
*Artyfechinostomum sufrartyfex* trematodes isolated from infected patients in Bihar, India. A) Trematodes in physiologic saline collected from stool samples. B) Whole mount of an adult trematode (acetocarmine stain). C) Metacercaria isolated from *Pila globosa* snails. Original magnification ×400. D*) Pila globosa* snails, the second intermediate host of the trematode.

To establish the source of infection, we attempted to identify the clinical parasite samples and the metacercariae. We morphologically identified representative parasite samples isolated from the patients (*2*,*4*,*5*). However, we could not identify metacercaria by only morphologic characteristics (Figure 2). Therefore, we used a molecular approach to confirm the identity of the life cycle stages.

We isolated total genomic DNA from individual adult trematodes (*6*). For metacercariae, we isolated genomic DNA from ≈200 cysts/isolation by using a DNA Isolation Kit (Macherey-Nagel, https://www.mn-net.com) according to the manufacturer’s protocol. We then amplified and sequenced nuclear 28S rRNA and internal transcribed spacer (ITS) 2 genes and the mitochondrial cytochrome c oxidase (COI) gene for both stages by using universal trematode primers (*7*–*9*). Individual gene regions tested were 1,042 bp for the 28S rRNA gene, 433 bp for ITS2 gene, and 343 bp for the mitochondrial COI gene. We deposited sequences in GenBank (accession nos. MH236132–3, MH237730–1, and MH253673–4).

For specific identification of the parasites, we performed a blastn search (https://blast.ncbi.nlm.nih.gov/Blast.cgi). The 28S rRNA and mitochondrial COI gene regions showed maximum sequence identity with GenBank accession no. KF781303.1 for *A. sufrartyfex* (99%) and accession no. NC037150.1 for *A. sufrartyfex* from Shillong, India (100%). For the ITS2 gene region, we observed maximum sequence identity with GenBank accession no. JF412727 *Echinostoma malayanum* from Khon Kaen, Thailand (99%), and with accession no. EF027100.1 *A. sufrartyfex* from Meghalaya, India (96%). On the basis of these findings, we concluded that clinical specimens and metacercariae isolated from *P. globosa* snails were the same species (*A. sufrartyfex*).

Furthermore, we generated barcode sequences by using trematode-specific primers (*10*) and deposited them in the BOLD database (BOLDSYSTEMS version, http://www.boldsystems.org). We obtained unique barcodes for both life cycle stages (identification no. BIN URI-BOLD:ADM2711). The barcode sequence had a length of 777 bp. To check for its specificity, we performed a similarity search across the BOLD database by using the BOLD Identification System. This search showed that our sequences were highly species specific; the closest match with other species was with *Nephrostomum limai* worms (83.24% identity).

## Conclusions

Our findings conclusively establish that these children were infected with *A. sufrartyfex* trematodes. We identified the causal agent and its infective metacercarial stage as *A. sufrartyfex* trematodes by using morphologic and molecular approaches. For ease of accurate identification in the future, we also provide unique DNA barcodes for the species.

Overall, we detected 170 infected case-patients and 11 deaths from these infections. Because of lack of proper diagnostic tools available to medical practitioners in the affected parts, several other infection cases might have remained undefined. This trematode species poses a serious threat to public health in this part of India and if not contained early, might spread to other and nonendemic areas of the region.

We also report the prevalence of trematode metacercariae in *P. globosa* snails from foci of infections in Bihar, thus implicating this snail species as the potential source of infection. At the same time, we found metacercaria prevalence to be quite high, which is indicative of greater transmission risk to the inhabitants who are eating raw snails.

We found the DNA barcodes generated for life cycle stages to be unique in the entire BOLD database. Therefore, we expect these barcodes to act as references for easy and accurate diagnosis of the disease in the future.

We observed that some high-risk practices, such as open defecation in the infected areas, are still rampant, which is a cause of concern because this practice helps maintain the parasite cycle in the environment. However, a cleanliness program, such as the Swachh Bharat Mission started by the Government of India (http://sbm.gov.in/sbmreport/home.aspx), and installing toilets in every household in rural areas might immensely help to contain the parasite infection.

Appendix Additional information on *Artyfechinostomum sufrartyfex* trematode infections in children, Bihar, India.
