# Supplementary material for: Artyfechinostomum sufrartyfex Trematode Infections in Children, Bihar, India
Source: Emerg Infect Dis. 2019 Aug;25(8):1571–3. doi: 10.3201/eid2508.181427 (PMC6649313; doi:10.3201/eid2508.181427)
Supplement: Appendix — Additional information on Artyfechinostomum sufrartyfex trematode infections in children, Bihar, India. [file 18-1427-Techapp-s1.pdf]

Article DOI: https://doi.org/10.3201/eid2508.181427

Artyfechinostomum sufrartyfex Trematode Infections in Children, Bihar, India

Appendix.

Appendix Table. Prevalence of *Artyfechinostomum sufrartyfex* trematode metacercaria in *Pila globosa* snails collected from foci of infections in 2 districts, Bihar, India*

Site no.	Site	District	GPS coordinates	No. snails collected	No. infected snails	Prevalence, %
1	Punaura	Sitamarhi	26°35′11.1″N, 85°27′39.8″E	456	220	48.24
2	Parsauni	Sitamarhi	26°31′3.3”N, 85°24′29.4″E	258	118	45.73
3	Hanumannagar	Sitamarhi	26°45′54.7″N, 85°34′50.1″E	62	10	16.12
4	Kacheripur	Sitamarhi	26°48′24.9″N, 85°35′69.0″E	269	67	24.90
5	Parsaunivaid	Sheohar	26°31′43.6″N, 85°19′81.7″E	234	54	23.07
6	Parori, Dumra	Sitamarhi	26°33′9.69″N, 85°30′47.52″E	177	54	30.50
7	Site 1, Dhankaul	Sitamarhi	26°31′34.3″ N, 85°27′39.8″E	179	46	25.69
8	Site 2, Dhankaul	Sitamarhi	26°31′34.3″ N, 85°27′39.8″E	206	63	30.58

*GPS, global positioning system.
